# Sexual Transmission of a Plant Pathogenic Bacterium, *Candidatus* Liberibacter asiaticus, between Conspecific Insect Vectors during Mating

**DOI:** 10.1371/journal.pone.0029197

**Published:** 2011-12-21

**Authors:** Rajinder S. Mann, Kirsten Pelz-Stelinski, Sara L. Hermann, Siddharth Tiwari, Lukasz L. Stelinski

**Affiliations:** Entomology and Nematology Department, Citrus Research and Education Center, University of Florida, Lake Alfred, Florida, United States of America; French National Centre for Scientific Research - Université Aix-Marseille, France

## Abstract

*Candidatus* Liberibacter asiaticus is a fastidious, phloem-inhabiting, gram-negative bacterium transmitted by Asian citrus psyllid, *Diaphorina citri* Kuwayama (Hemiptera: Psyllidae). The bacterium is the presumed causal agent of huanglongbing (HLB), one of the most destructive and economically important diseases of citrus. We investigated whether Las is transmitted between infected and uninfected *D. citri* adults during courtship. Our results indicate that Las was sexually transmitted from Las-infected male *D. citri* to uninfected females at a low rate (<4%) during mating. Sexual transmission was not observed following mating of infected females and uninfected males or among adult pairs of the same sex. Las was detected in genitalia of both sexes and also in eggs of infected females. A latent period of 7 days or more was required to detect the bacterium in recipient females. Rod shaped as well as spherical structures resembling Las were observed in ovaries of Las-infected females with transmission electron microscopy, but were absent in ovaries from uninfected *D. citri* females. The size of the rod shaped structures varied from 0.39 to 0.67 µm in length and 0.19 to 0.39 µm in width. The spherical structures measured from 0.61 to 0.80 µm in diameter. This investigation provides convincing evidence that a plant pathogenic bacterium is sexually transmitted from male to female insects during courtship and established evidence that bacteria persist in reproductive organs. Moreover, these findings provide an alternative sexually horizontal mechanism for the spread of Las within populations of *D. citri*, even in the absence of infected host trees.

## Introduction

*Candidatus* Liberibacter asiaticus (Las) is a fastidious, phloem-inhabiting, gram-negative bacterium transmitted by Asian citrus psyllid, *Diaphorina citri* Kuwayama (Hemiptera: Psyllidae). The bacterium is the putative causal pathogen of huanglongbing (HLB) disease of citrus; however, Koch's postulates have not yet been fulfilled [1]–[2]. HLB is one of the most destructive and economically important diseases of citrus worldwide [1], [3]. HLB causes severe yield reduction, fruit drop, reduced fruit quality, and tree death. Currently, there is no cure for the disease. Two related species, *Candidatus* L. americanus (Lam) and *Candidatus* L. africanus (Laf), are also presumed casual agents of the disease [4]–[6]. Until recently, Lam was found only in Brazil but has now been reported in Hunan, China [6]. Laf is the presumed causal agent of HLB in South Africa, Saudi Arabia, and on a few islands in the Indian Ocean [6]. *Diaphorina citri* is the insect vector of Las in Asia, Brazil and U.S.A., whereas *Trioza erytreae* (Del Guercio) (Triozidae) is the vector of Laf [1], [6], [7].

Las is acquired more efficiently by psyllid nymphs than adults [8], [9], [10]. Although up to 100% of *D. citri* reared on infected citrus may acquire Las, acquisition by adults averaged 40% following 5 weeks of feeding on infected plants [10]. Furthermore, successful transmission, as measured by plant infection, by individual *D. citri* may range from 4 to 10% annually, following inoculations [10]. Certain studies have suggested that Las persists and replicates within the vector following acquisition [8], [9]. In addition to acquisition from infected host plants, Las is transmitted transovarially at a low rate (2–6%) from infected females to their offspring [10]. To date, sexual transmission of plant pathogenic bacteria among mating pairs of insects has not been investigated.

Several arthropod viruses and protozoa are sexually transmitted between partners, (reviewed by Knell and Webberley, [11]). Insect-transmitted animal and plant viruses such as the Dengue Fever virus, St. Louis Encephalitis virus, Tomato yellow leaf curl virus, Squash leaf curl virus, and Watermelon chlorotic stunt virus are transmitted during mating [11]–[13]. Sexual transmission of these viruses is presumably an adaptation that enables horizontal spread within the vector population and may be an important factor in maintaining these viruses in their vector populations if plant hosts are absent or vertical transmission is inefficient [14]–[15].

Although many insect bacteria are known to be vertically transmitted from mother to offspring, sexual transmission of bacteria between partners has rarely been reported in insects [11], [16]. Sexual transmission of the endosymbiotic bacteria, *Candidatus* Hamiltonella defensa and *Candidatus* Regiella insecticola, was recently described in the pea aphid, *Acyrthosiphon pisum* [17]. Similarly, sexual transmission of symbiotic bacteria belonging to the genus *Asaia* was demonstrated in *Anopheles stephensi* mosquitoes [16]. *Wolbachia* spp. have been recorded in the testes and ovaries of male and female insects, which suggested the possibility of sexual transmission; however, these are exclusively vertically transmitted via egg cytoplasm and are not transmitted to females during mating [18]. The absence of bacterial transmission from males to females in *Drosophila melanogaster* during mating is attributed to the production of a peptide with anti-microbial properties, termed “andropin”. Andropin is presumed to be manufactured in the testes of male *D. melanogaster* and passed to females during mating to prevent sperm degradation [11], [19], [20]. The protein is secreted into female accessory fluid during copulation [11]. However, Otti et al. [21] did not find andropin in male ejaculates of the bed bug, *Cimex lectularius*, but suggested male sperm protection through production of bacteriolytic lysozymes. Although sexual transmission of endosymbiotic bacteria is known to occur as described above, sexual transmission of insect-transmitted plant pathogenic bacteria has not been reported previously. Herein, we report sexual transmission of Las from infected male *D. citri* to females during mating.

## Materials and Methods

### Maintenance of insects, pathogen and host plants

Uninfected adult *D. citri* used in bioassays were obtained from a laboratory culture continuously reared at the University of Florida Citrus Research and Education Center (Lake Alfred, USA). The culture was established in 2000 from field populations in Polk Co, FL, U.S.A. (28.0′N, 81.9′W) prior to the discovery of HLB in the State. Insects were maintained on sour orange (*Citrus aurantium* L.) and ‘Hamlin’ orange [*C. sinensis* (L.) Osb.] at 27±1°C, 63±2% RH, and a 14∶10 L∶D photoperiod. Monthly testing of randomly sampled *D. citri* nymphs and adults was conducted using quantitative real-time polymerase chain reaction (qPCR, described below) to confirm that psyllids did not harbor Las. *Diaphorina citri* harboring Las were obtained from Las-infected *C. aurantium* and *C. sinensis* plants maintained in a secure quarantine facility under the same environmental conditions used for rearing the uninfected *D. citri* culture. The Las-infected culture originated from the uninfected culture. Routine sampling indicated that approximately 40% of *D. citri* individuals obtained from the infected culture were positive for Las when groups of 10–20 psyllids were tested with qPCR. Uninfected and Las-infected *D. citri* cultures were maintained in separate rearing facilities to preclude cross contamination.

Cultures of Las in plants were maintained by graft-inoculating healthy *C. sinensis* with Las-infected budwood collected from commercial citrus groves in Immokalee, FL (Collier Co.). *Bergera koenigii* plants were obtained as potted seedlings from an HLB-free local nursery. The plants were confirmed negative for Las infection with qPCR. Uninfected and Las-infected plants were maintained in separate, secure greenhouses to minimize the likelihood of cross contamination.

### Detection of Las in insect and plant samples using qPCR

Dual-labeled probes were used to detect Las in *D. citri* and plants using an ABI 7500 qPCR system (Applied Biosystems, Foster City, CA) in a multiplex TaqMan qPCR assay previously described by Li et al. [22] and Pelz-Stelinski et al. [10]. DNA from insect and plant samples was isolated using the DNeasy blood and tissue or DNeasy plant kits (Qiagen Inc, Valencia, CA), respectively. The target primers LasF (5′-TCGAGCGCGTATGCAATACG-3′), LasR (5′-GCGTTATCCCGTAGAAAAAGGTAG-3′) and the probe HLBp (FAM-AGACGGGTGAGTAACGCG-BHQ1) were used for amplifications of Las-specific 16S rDNA from psyllid and plant extracts. Reactions were conducted using probe-primer sets targeting internal control sequences specific to *D. citri* [insect *wingless*: WgF (5′-GCTCTCAAAGATCGGTTTGACGG-3′), WgR (5′-GCTGCCACGAACGTTACCTTC-3), WGp (FAM- TTACTGACCATCACTCTGGACGC –BHQ2)] or plant [cytochrome oxidase: CoxF (5′-GTATGCCACGTCGCATTCCAGA-3′), CoxR (5′-GCCAAAACTGCTAAGGGCATTC-3′), and COXp (FAM- ATCCAGATGCTTACGCTGG –BHQ2)] gene regions [10], [22], [23]. All primers and probes were obtained from Integrated DNA Technologies (Coralville, IA). Psyllid PCR reactions contained 1 µl of DNA template, 12.5 µl of TaqMan Universal PCR Master Mix (Applied Biosystems), 235 nM of each of target primer, and 118 nM each of Wg and Las probes. Plant reactions were conducted similarly, with 216 nM of Las primers, 270 nM internal control primers, and 135 nM of each probe. Duplicate PCR reactions for each sample were performed in 96-well MicroAmp reaction plates (Applied Biosystems, Carlsbad, CA,) using an initial denaturation step of 95°C for 10 min, followed by 40 cycles of 95°C for 15 s and 60°C for 60 s. In addition to samples, each plate also contained a no template (negative) control, a positive control for Las, and a positive control for psyllid or plant DNA. Reactions were considered positive for either target sequence if the cycle quantification (Cq) value, determined by the ABI 7500 Real-Time software (version 1.4, Applied Biosystems), was ≤36.

### Sexual transmission assays

To determine whether Las is transmitted sexually between *D. citri* during mating, the following treatments were established: 1) infected (Las+) male and uninfected (Las−) female; 2) Las+ female and Las− male; 3) Las+ female and Las− female; 4) Las+ male and Las− male; 5) Las− female and Las− male (negative control) and; 6) Las+ female and Las+ male (positive control). For each treatment, psyllids were enclosed in plastic Petri dishes (60 mm diam) (Fisherbrand, Thermo Fisher Scientific, Waltham, MA) containing 1.5% agar to form a solidified bed. Each solidified agar bed was covered with Whatman no. 1 filter paper (Whatman Inc, Piscataway, NJ) to prevent insects from sticking to the agar. Psyllids were provided with 10% sugar solution enclosed within two stretched Parafilm strips. The sucrose-solution artificial diet was employed to preclude transmission of the Las pathogen between psyllids due to feeding. For each treatment combination, four pairs of sexually mature (>3 days old, Wenninger and Hall [24]) adult psyllids were enclosed in Petri dishes and held at 25±1°C, 50±5% RH and a 14∶10 (L∶D) photoperiod for a 72 h mating period. In treatments with same sex couples, infected or uninfected psyllids were marked with a florescent dot on the thorax to identify the potential donor of Las. This marking procedure was rotated and randomized between infected and uninfected psyllids. After 72 hr, psyllids were collected and stored in 80% ethanol at −20°C for subsequent DNA extraction and Las detection with qPCR, as described above. Each treatment combination was replicated 20 times comprising four donor and four recipient insects per replication. The entire experiment was repeated twice on different dates resulting in 160 donors (initially positive) and 160 recipient (initially negative) insects for each treatment combination.

A second experiment was conducted that evaluated the same treatment combinations described above. However, the objective was to include a latent period following mating to allow more time for potential bacterial replication. Psyllid pairs were confined in agar-filled Petri dishes for 72 hr to allow mating as described above. Thereafter, psyllids were segregated and recipient insects were reared for an additional 7 or 14 days to allow time for potential multiplication of sexually transmitted Las. During this latent period, psyllids were enclosed within mesh sleeve cages on *B. koenigii* plants, which are hosts for *D. citri* but not Las [25]. All *B. koenigii* test plants were analyzed for Las infection four months after experiments were conducted to ensure that they had not become infected with Las. Donor and recipient psyllids were then stored in 80% ethanol at −20°C prior to qPCR detection of Las. The entire experiment was replicated twice on different dates, with each treatment combination replicated 20 times (n = 160 per combination).

### Detection of Las in *D. citri* genitalia

The objective of this experiment was to determine if Las was present in testis or ovaries of male or female Las-infected *D. citri*, respectively. Male and female *D. citri* adults reared on Las-infected citrus plants were anesthetized by freezing at −20°C for ≈5 min. Anesthetized adults were dissected in 1× PBS 10 mM phosphate buffer saline (PBS, pH 7.4) using a steel surgical dissecting knife (Fisher Scientific, Waltham, MA) under a Wild MC3 stereomicroscope (Leica, Heerbrugg, Switzerland). Las infection in dissected adults was subsequently confirmed with qPCR. Genitalia were extracted and successively washed with 70% ethanol, 10% sodium hypochlorite (Clorox®, regular bleach), and sterile 1× PBS to remove hemolymph-derived Las contamination prior to storage in 80% ethanol at −20°C. Ovaries and testis obtained from adults that tested positive were further analyzed by qPCR for Las infection in groups of one or five specimens. For males, the testis and adeagus were analyzed simultaneously because of their small size and to avoid loss of bacteria during dissection. In total, 34 male and 32 female individual genital samples obtained from confirmed Las-positive *D. citri* adults were analyzed for each sex. Twenty specimens for each sex were analyzed when the genitalia were tested in groups of five. An equal number of genital samples obtained from confirmed Las-negative *D. citri* adults were used as a negative control.

### Detection of sexually-transmitted Las in unlaid eggs of *D. citri* females which acquired Las during mating

The objective of this experiment was to determine if Las was present in unlaid eggs of female *D. citri*, which acquired the bacterium from males during mating. In this experiment, uninfected females were mated with presumably Las-positive males in Petri dishes to obtain Las-positive females using the above-described procedures. Uninfected females mated with uninfected males were used as a negative control. Las-positive females mated with Las-positive males were used as a positive control. Each treatment combination was replicated 80 times comprising four donor and four recipient insects per replication. After 3 days, females that presumably acquired Las sexually were removed from the Petri dishes and released onto *B. koenigii* plants housed within mesh sleeve cages. Seven days later, female *D. citri* were collected and dissected in 1× PBS to extract ovaries and unlaid eggs. Eggs were successively washed with 70% ethanol, 10% sodium hypochlorite, and sterile 1× PBS to remove hemolymph-derived Las contamination. This procedure was verified in preliminary testing to remove potential Las contamination and our negative and positive control treatments (see Results) corroborate that contamination did not occur. Extracted egg samples were stored in 80% ethanol at −20°C for subsequent qPCR analysis. Las infection in dissected adults (from which eggs were extracted) was confirmed with qPCR as described above. Unlaid eggs (n = 180) obtained from females that acquired bacteria from males (n = 9) were further analyzed for Las infection. Eggs were analyzed for Las infection in groups of 20 specimens resulting in 9 egg samples. Egg samples for the negative control and positive control treatments comprised 20 samples in groups of 20 specimens.

### Detection of sexually-transmitted Las in F1 offspring of females that acquired Las during mating

To determine if females that acquired Las during mating could subsequently transmit the bacterium transovarially, sets of four sexually mature, Las-negative female *D. citri* were confined with Las-positive males in Petri dishes to allow mating. Uninfected females confined with uninfected males were used as a negative control. In total, there were 40 Petri dishes for each treatment with each Petri dish representing a replicate. After 3 days, females were removed from Petri dishes and released onto *B. koenigii* plants for 7 days to allow egg laying in batches of 20 insects per plant resulting in eight replicates for each treatment. The eggs were allowed to develop and emerging adults (F1) were analyzed for Las infection using qPCR. The parent male and female psyllids were stored in 80% ethanol at −20°C and also analyzed for Las infection with qPCR. All *B. koenigii* plants were analyzed for Las infection four months after the experiment was conducted to ensure they had not become infected with Las.

### Transmission Electron Microscopy (TEM) of ovaries from Las-infected females

Ovaries of Las-infected *D. citri* were inspected by TEM to determine whether they harbored structures that resemble Las morphologically. Twenty *D. citri* females, presumably infected with Las, were examined. These psyllids were obtained from the Las-infected culture described previously. As a negative control, twenty uninfected females reared on healthy citrus plants were also examined. Insects were anesthetized by freezing at −20°C for ≈5 min, then decapitated using a steel surgical dissecting knife. Ovaries were removed from females under 40× magnification using a Wild MC3 dissecting microscope, then subjected to disinfecting and washing as described above. Samples were prepared for TEM as in Onagbola et al. [26]. Briefly, individual ovaries were fixed in 3% glutaraldehyde in 0.1 M potassium phosphate buffer (pH 7.2) for 24 h at 4°C. After rinsing in the buffer solution, samples were post fixed in 1% osmium tetroxide for 2 h, washed in 2× phosphate buffer, then fixed in 2% osmium tetroxide for 6 h at room temperature. The tissues were dehydrated in 10, 20, 30, 100% acetone for 0.25, 0.25, 0.25, and 3 h, respectively, and then infiltrated in 30, 50, 70, and 100% Spurr's resin for 1, 8, 14, and 14 h, respectively. Samples were embedded in pure Spurr's plastic for 24 h, placed in molds, covered with fresh resin, and oven dried at 70°C for 24 hr. Polymerization was performed at 70°C for 72 hr. Ultrathin sections of the tissues were cut with a diamond knife on an ultramicrotome (model 8365, LKB-Huxley Cambridge, United Kingdom) and placed on Gilder grids previously coated with 0.5% formvar (Electron Microscopy Sciences, Fort Washington, PA) solution. Blocks were sectioned, stabilized in 2% uranyl acetate, and placed on 0.5% formvar-coated nickel grids (Electron Microscopy Sciences, Fort Washington, PA, 200 mesh) and stained with uranyl-acetate (2% aqueous, 20 min) and lead citrate (6 min). Grids with the affixed ultrathin sections were cleared by submersing in lead citrate to regulate electron density. Sections were rinsed with 2% sodium hydroxide solution followed by excess distilled water before examination under a Morgagni #268 transmission electron microscope (FEI Electron Optics, Einhoven, The Netherlands) at 60 kV.

### Transmission of Las to healthy plants following sexual transmission

An experiment was conducted to test whether female *D. citri* that obtained Las through sexual transmission from infected males would subsequently transmit the bacterial pathogen to uninfected citrus plants by feeding. Las-negative female *D. citri* were confined with Las-positive males in Petri dishes to allow mating as described above. Uninfected females confined with uninfected males were used as a negative control. After 3 days, females were removed from Petri dishes and released onto 2–3 month-old, uninfected (confirmed by qPCR) grapefruit plants (*Citrus paradesi*) and allowed to feed for a 7-day inoculation access period (IAP). In total, each treatment was replicated 10 times (10 plants) and 10 female *D. citri* were released per replicate plant following mating. The entire experiment was replicated twice. Following 7 day IAP, all living *D. citri* were collected, freeze killed and placed individually into sterile 1.5 mL centrifuge tubes containing 80% ethanol and stored at −20°C until DNA extraction. All plants were maintained in Plexiglass cages for eight months as described above for plant maintenance. Plants were sampled (as described above) for Las infection at 4 and 8-month intervals following IAP to determine infection status. Male and female *D. citri* were also tested for Las infection directly following mating and IAP, respectively, using the qPCR methods described above.

### Data Analysis

Logistic regression with Firth's bias reduction was used to analyze the data from all experiments. The data from the sexual transmission assays were analyzed using mating pair treatment and post-mating latent period as independent variables and infection status of the recipient insect as the dependent variable (PROC LOGISTIC, SAS Institute, Cary, NC). Duration of post-mating period did not significantly impact the infection status of recipient insects. Therefore, data from post mating periods were pooled for further analysis. Data from experiments evaluating Las in genitalia were also analyzed using logistic regression with Firth's bias reduction using source of genitalia, gender, and grouping as independent variables and infection status of genitalia as a dependent variable. In experiments evaluating transovarial transmission, mating pairs were used as an independent variable, while infection status of eggs or F1 offspring was used as a dependent variable.

## Results

### Sexual transmission assays

In the first experiment, an infection rate of 44% was observed in donor Las-infected male or female *D. citri* paired with uninfected male or female partners. For all mating pair treatments, Las was not detected in recipient male or female *D. citri* collected immediately after the 72 h mating period.

In contrast, mating pair treatment type significantly affected infection status of recipient insects when the same mating pairs were provided with a post-mating period (χ^2^ = 98.81, df = 5, p<0.01). When infected males (donor insect) were mated with uninfected females (recipient insect), 3.8% of the recipient females acquired Las from donor males (Table 1). Las was not detected in recipient male or female insects which were confined with donor insects of the same sex. Las was also not detected in *B. koenigii* plants four months after experiments were conducted.

**Table 1:** Venereal transmission of Las between adult *D. citri*.

Mating pair treatment	Percent of donor insects that were infected with Las	Percent of recipient insects that became infected with Las after mating	3Odds ratios of recipient insect acquiring Las over negative control
Las-positive males mated with Las-negative females	43.8^1^ (0.04)^2^	3.8 (0.01)	13.5
Las-negative males mated with Las-positive females	42.5 (0.04)	0.0 (0.00)	1
Las-negative females mated with Las-positive females	38.8 (0.04)	0.0 (0.00)	1
Las-negative males mated with Las-positive males	37.5 (0.03)	0.0 (0.00)	1
Las-negative males mated with Las-negative females (negative control)	0.0 (0.00)	0.0 (0.00)	1
Las-positive males mated with Las-positive females (positive control)	40.6 (0.04)	45.0 (0.04)	262.9

1 Samples were considered positive if the Cq values were ≤36.

2 Values in parenthesis are standard error of means.

3 Odds (ratios of probabilities) of detecting Las in a particular mating pair treatment over the odds of detecting Las in the negative control. An odds ratio = 1.0 indicates equal chance of detecting Las between treatment and control.

### Detection of Las in *D. citri* genitalia

Source of genitalia significantly affected the presence of Las in *D. citri* genitalia (χ^2^ = 6.03, df = 1, p = 0.01). Las was detected in 13.5% of genital samples when genitalia were extracted from Las-positive pyllids (Table 2). No Las was detected in the genetalia of psyllids from the uninfected culture (Table 2). The odds (ratios of probabilities) that genitalia were positive for Las was 31.4 times greater when samples were extracted from Las-positive than negative psyllids. Las was detected in 5.9% of male and 12.5% of female genital samples from individual Las-infected *D. citri* (Table 2). The odds of Las infection was 2.4 times greater for female than male genitalia. However, Las infection was statistically equivalent in the genitalia of males and females. When the genitalia from Las-infected psyllids were pooled in groups of five insects, 10.0% of male and 25.0% of female genital samples tested positive for Las (Table 2). The odds of detecting Las from pooled genital samples were 2.1 times greater than from individual samples. However, there was no significant effect of pooling samples on Las infection in *D. citri* genitalia.

**Table 2:** Infection status of *D. citri* genitalia from Las-positive and negative cultures.

		Percent of *D. citri* genital samples that were infected with Las		
Source of *D. citri* genitalia^1^	*D. citri* gender	Individual genital samples	Pooled from five genital samples	Combined mean^4^
**Confirmed Las-positive insects**	**Male**	5.9^2^ (0.04)^3^	10.0 (0.07)	
	**Female**	12.5 (0.03)	25.0 (0.10)	13.48a
**Confirmed Las-negative insects**	**Male**	0.0 (0.00)	0.0 (0.00)	
	**Female**	0.0 (0.00)	0.0 (0.00)	0.0b
**Mean**	**-**	9.2	17.5	-

1 Genitalia were secured from confirmed Las-positive or Las-negative *D. citri*.

2 Samples were considered positive if the Cq values were ≤36.

3 Values in parenthesis are standard error of means.

4 Combined mean percent of Las infection in male and female *D. citri* derived from combined individual and pooled samples.

Values within columns labeled with different letters are significantly different (p<0.05).

### Detection of sexually-transmitted Las in unlaid eggs of *D. citri* females that acquired Las during mating

In this experiment, 2.8% of the initially uninfected females acquired Las from infected males during mating (Table 3). All unlaid egg samples from females that acquired Las from infected males were negative (Table 3). There were no significant differences between the treatments tested for Las infection; however, 5.0% of unlaid egg samples obtained following matings of Las-positive males with Las-positive females (positive control) were positive for Las (Table 3). For the negative control, Las was not detected in eggs of donor or recipient insects (Table 3).

**Table 3:** Infection status of unlaid eggs from *D. citri* females that acquired Las during mating.

	Percent Las infection following mating	
Mating pair treatment	Entire recipient female insects analyzed	Unlaid eggs only from recipient females
Las-positive males mated with Las-negative females	2.8^1^ (0.01)^2^	0.0 (0.00)
Las-positive males mated with Las-positive females	44.7 (0.03)	5.0 (0.05)
Las-negative males mated with Las-negative females	0.0 (0.00)	0.0 (0.00)

1 Samples were considered positive if the Cq values were ≤36.

2 Values in parenthesis are standard error of means.

### Detection of sexually-transmitted Las in F1 offspring of females that acquired Las during mating

In this experiment, 5.6% of the initially uninfected parent females acquired Las from infected males during mating. None of the uninfected female *D. citri* confined with uninfected males (negative control) tested positive for Las. There was no significant difference between the number of F1 adults emerging from matings of Las-positive males with Las-negative females and those emerging from Las-negative females mated with Las-negative males. Las was detected in 0.8% of F1 adults emerging from eggs that resulted from matings between Las-positive males with Las-negative females. No Las was detected in F1 adults emerging from the negative control treatment.

### Transmission Electron Microscopy (TEM) of ovaries from Las-infected females

**Figure 1:**
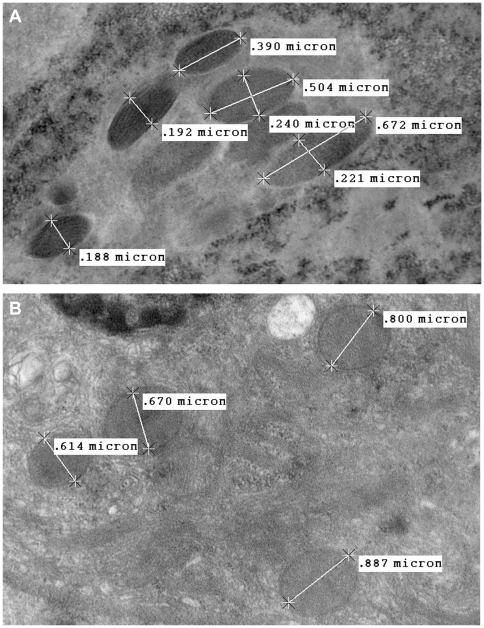
Rod shaped (A) and spherical (B) structures resembling Las in ovaries of Las-infected female *D. citri* observed with transmission electron microscopy. Ovaries from female psyllids that were confirmed negative for Las by qPCR were devoid of structures similar to those observed from positive females following an identical microscopic evaluation.

Rod-shaped as well as spherical structures resembling Las were observed under TEM in the ovaries extracted from females that tested positive for Las with qPCR (Figure 1 A,B). Rod-shaped structures varied in size from 0.39 to 0.67 µm in length and 0.19 to 0.39 µm in width (Figure 1 A). The spherical structures measured 0.61 to 0.80 µm in diameter (Figure 1 B). Ovaries from female psyllids that were confirmed negative for Las by qPCR were devoid of structures similar to those observed from positive females following an identical microscopic evaluation.

### Transmission of Las to healthy plants following sexual transmission

At 4 and 8 months following a 7-day IAP by initially uninfected female *D. citri* that were mated with presumably infected male *D. citri*, none of the initially healthy citrus plants (*Citrus paradesi*) tested were positive for Las. Approximately 43% of donor males were infected with Las in this experiment and 2.3% of recipient females were infected with Las post mating.

## Discussion

Detection of Las in female psyllids reared on Las-free citrus plants after mating with infected males strongly suggests that this plant pathogenic bacterium was transferred sexually from male to female insects during mating. This was also confirmed with lack of transmission from females to males, or between same sex pairs. While this phenomenon has been demonstrated experimentally for endosymbiotic bacteria of insects, this is the first report of sexual transmission of plant pathogenic bacteria among insect vectors.

Las presumably persists and multiplies within *D. citri* following acquisition [9], [27]. This may explain why Las was not detected in recipient females immediately after mating with infected males, but instead required a seven-day or longer latent period prior to detection. It is possible that only a small number of bacteria are passed from males to females during mating, which must then replicate within the psyllid before reaching a sufficient titer for detection by qPCR. These results are congruent with those of Pelz-Stelinski et al. [10], who reported a one-week latent period prior to detection of Las by identical qPCR methods following feeding on Las-infected plants.

The distribution of Las appears to be ubiquitous throughout the hemolymph and organs of infected psyllids, although the bacterial titer is highest in the alimentary canal and salivary glands [28]. We observed a low bacterial titer (Cq values 37 to 39) in 2.3% and 8.9% of individual male and female genital samples, respectively (data not shown), suggesting a low bacterial titer in the reproductive organs of *D. citri*. Cq values could also be high because of the small amount of tissue that is processed during DNA extraction even though there may be a high bacterial load relative to the size of the organ examined. If the overall titer of bacteria in the infected psyllid donor is low, the likelihood of bacteria occurring in the reproductive organs should also be correspondingly low.

In the current investigation, the rate of Las transmission from infected males to uninfected females was low (3.8%). In addition to low bacterial titer, several other factors could contribute to the low rate of Las transmission to females, including the timing of mating relative to oviposition, insect age, interactions between the pathogen and other psyllid symbionts, or physiological responses of psyllids to the pathogen [11]. In particular, the presence of antibacterial peptides may reduce Las titer in the sperm of infected male psyllids. Antibacterial peptides, which occur in sperm of certain insect species, function to protect sperm from degradation by pathogenic bacteria [11], [20], [21]. However, reports of sperm protection are limited to andropin in *D. melanogaster* and bacteriolytic lysozymes in *Cimex lectularius* and have not been documented in other insect species. Furthermore, antibacterial peptide production is a specific response induced by contact with pathogenic bacteria [29]. If similar peptides were identified in association with male psyllids, it is unclear whether Las would be recognized as pathogenic.

Presence of bacteria in male genitalia implicates their transfer through sperm and/or other seminal fluids during mating as the mechanism of venereal transmission. During copulation, male insects transfer sperm to females, as well as, a variety of other substances including seminal proteins and peptides produced in accessory glands [30]. The components and quantity of ejaculate or seminal fluids transferred from male to female *D. citri* have not been investigated to date. Traumatic insemination or wounding is also possible during mating; however, it is an unlikely mechanism by which Las may be transmitted from males to females. Such mating behavior has not been documented for *D. citri* and female to male or same sex transmissions were not observed.

Both rod shaped (0.4 to 0. 7 µm in length and 0.2 to 0.4 µm in width) and spherical (0.61 to 0.80 µm in diameter) structures were observed in ovaries of females that were infected by Las (Figure 1 A,B). These structures were morphologically similar in size and appearance to previously reported rod shaped (0.6 to 1.2 µm in length and 0.2 to 0.8 µm in width) and spherical (0.1 to 0.2 µm in diameter) structures identified as Las in citrus plant tissues [31]–[35]. However, the identity of these structures requires confirmation by molecular assays. Fluorescent *in situ* hybridization (FISH) would be an optimal technique to further confirm the identity of bacteria associated with female *D. citri* ovaries; however, this is currently not possible given that Las remains recalcitrant to culturing [34]. Until this occurs, non-specific probes targeting bacterial 16S rRNA coupled to immunogold or fluorescent labels remain the only options for targeting Las in psyllids. Although FISH would confirm the presence of bacterial cells, this technique would not provide further taxonomic clarification of the identity of bacteria as compared with TEM.

Detection of Las in the unlaid eggs and offspring of infected females is congruent with previously reported transovarial transmission of Las by *D. citri* [10]. Las was not detected in egg samples collected from recipient females that acquired Las from infected males. The small sample size available for testing likely contributed to the lack of positive egg samples from this treatment. Transovarial transmission of Las by *D. citri* is reported to occur at a rate of 3.6%. The current results indicate that less than 6% of females acquired the bacteria sexually; hence, only a small percentage of these females could be expected to transmit Las transovarially. In addition, it was necessary to pool eggs from infected females for analysis, as the titer of DNA present in individual eggs is insufficient for qPCR analysis [27]. Pooling eggs for pathogen detection may have diluted bacterial titer from Las-positive eggs within the samples tested, resulting in a bacterial titer below detectable limits. Detection of Las in F1 offspring of females that acquired Las during mating further confirmed that venereally acquired Las is subsequently transmitted transovarially.

Sexual transmission of endosymbiotic bacteria between insects during mating has been reported previously [11], [16], [17]. The relationship between *Candidatis* Liberibacter spp. and psyllid vectors is presumed to be symbiotic, with Las acting both as a phloem limited plant pathogen and as an insect symbiont [36]–[39]. It remains unclear whether the pathogen evolved within the psyllid vector or its host plants; however, recent studies examining the fitness of infected psyllids suggest that the pathogen-vector relationship is a well-adapted mutualism [Pelz-Stelinski et al. unpublished results]. Sexual transmission of Las provides additional, albeit indirect, evidence that the relationship between the pathogen and *D. citri* could be mutualistic rather than pathogenic. However, the potential benefits *D. citri* receives from Las infection require further investigation.

Sexual transmission of Las may confer similar benefits to those associated with transovarial transmission. The success of plant pathogen propagation should be higher if horizontal and vertical mechanisms of transmission exist within populations of insect vectors, particularly during periods when host plant species are absent or in limited supply. This may be particularly true for Las, for which *D. citri* is the only known vector [1]. *D. citri* can survive and, in some cases, reproduce on a range of Rutaceous host plant species that vary in their susceptibility to Las infection [1], [25], [40], [41]. Transfer of Las during mating and through transovarial transmission ensures that populations of Las survive until a favorable plant host becomes available. Sexual transmission of plant pathogens may be a particularly beneficial adaptation for maintaining their populations within insect vector populations when vertical transmission is low or completely absent [14]–[15]. Inoculation of plants by females that acquired Las sexually was not observed in the current study. Given that the probability of Las inoculation into a citrus plant by a single psyllid is 0.062 [10] and the probability of sexual transmission between infected males and uninfected females is 0.038 [current results], the combined probability of successful inoculation of a plant by a single female *D. citri* that acquired Las sexually would be 2×10^−3^. Therefore, approximately 500 uninfected female psyllids would need to be mated with known Las-infected males to result in inoculation of a single host plant as per the methods used in the current investigations. Assuming an average male infection rate of 40%, 1,250 individual mating experiments between pairs of male and female psyllids would be required to obtain a single infected plant. Given the number of sexual transmissions we were logistically capable of conducting, this likely explains why we were unable to document subsequent transmission of Las to plants by infected female insect vectors that acquired Las from males during copulation in the current investigation. Although, the overall percentage of sexual transmission of Las from males to females appears low, it could be a highly significant contributing factor to pathogen spread given that thousands of psyllids colonize individual trees. Up to 360 *D. citri* adults can be captured per sticky trap per tree per week and hundreds to thousands of immature eggs and nymphs can be laid be per tree per week [42].

Collectively our results demonstrate that Las is transmitted from male to female *D. citri* at a low rate during mating. These results suggest that the presumed causal agent of HLB may spread within populations of the vector even in absence of infected host plants. Further investigations are necessary to determine the role of sexual transmission in the epidemiology of HLB disease spread in citrus.
